# 3-Benzyl-1-methyl­imidazolium picrate

**DOI:** 10.1107/S1600536809035454

**Published:** 2009-09-09

**Authors:** Min Pi, Xiu-Ling Liu, Ji-Jun Xu, Chuan-Ming Jin

**Affiliations:** a Hubei Key Laboratory of Pollutant Analysis & Reuse Technology, College of Chemistry and Environmental Engineering, Hubei Normal University, Huangshi, Hubei 435002, People’s Republic of China

## Abstract

In the title salt, C_11_H_13_N_2_^+^·C_6_H_2_N_3_O_7_^−^, the dihedral angles between the benzene ring in the cation and the imidazolium ring and the benzene ring of the picrate anion are 113.7 (2) and 116.3 (2)°, respectively. The imidazolium ring is nearly parallel to the benzene ring of the picrate anion, the dihedral angle between the planes being 2.6 (1)°. The nitro groups in the picrate anions are disordered (occupancy ratio 0.54:0.46). The crystal packing is stabilized by weak C—H⋯O inter­actions between the cation–anion pairs.

## Related literature

For civilian and military applications of energetic materials, see: Sikder & Sikder (2004 ▶). Heterocyclic organic salts with low melting points are a new class of energetic materials, which have attracted considerable inter­est because of their ‘green chemistry’ properties, see: Singh *et al.* (2006 ▶). Picric acid is a polynitro­gen compound with explosive character and imidazolium-based cation picrate salts are good candidates for energetic ionic salts, see: Jin *et al.* (2005 ▶).
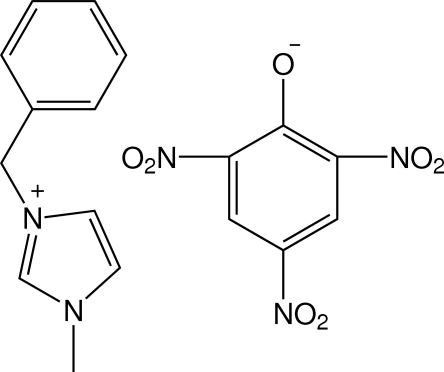


## Experimental

### 

#### Crystal data

C_11_H_13_N_2_^+^·C_6_H_2_N_3_O_7_^−^
*M* *_r_* = 401.34
Triclinic, 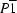

*a* = 9.1322 (6) Å
*b* = 10.2060 (7) Å
*c* = 10.8744 (7) Å
α = 63.6190 (10)°
β = 80.1660 (10)°
γ = 86.4820 (10)°
*V* = 894.52 (10) Å^3^
*Z* = 2
Mo *K*α radiation
μ = 0.12 mm^−1^
*T* = 298 K
0.20 × 0.10 × 0.10 mm

#### Data collection

Bruker SMART APEX CCD area-detector diffractometer
Absorption correction: multi-scan (**SADABS**; Sheldrick, 1996 ▶) *T*_min_ = 0.986, *T*_max_ = 0.988
5623 measured reflections
3447 independent reflections
2610 reflections with *I* > 2σ(*I*)
*R*_int_ = 0.046

#### Refinement

*R*[*F* ^2^ > 2σ(*F* ^2^)] = 0.056
*wR*(*F* ^2^) = 0.144
*S* = 1.04
3447 reflections
320 parameters
15 restraints
H-atom parameters constrained
Δρ_max_ = 0.35 e Å^−3^
Δρ_min_ = −0.24 e Å^−3^

### 

Data collection: *SMART* (Bruker, 2001 ▶); cell refinement: *SAINT-Plus* (Bruker, 2001 ▶); data reduction: *SAINT-Plus*; program(s) used to solve structure: *SHELXS97* (Sheldrick, 2008 ▶); program(s) used to refine structure: *SHELXL97* (Sheldrick, 2008 ▶); molecular graphics: *SHELXTL* (Sheldrick, 2008 ▶); software used to prepare material for publication: *SHELXTL*.

## Supplementary Material

Crystal structure: contains datablocks I, global. DOI: 10.1107/S1600536809035454/jj2007sup1.cif

Structure factors: contains datablocks I. DOI: 10.1107/S1600536809035454/jj2007Isup2.hkl

Additional supplementary materials:  crystallographic information; 3D view; checkCIF report

## Figures and Tables

**Table 1:** Hydrogen-bond geometry (Å, °)

*D*—H⋯*A*	*D*—H	H⋯*A*	*D*⋯*A*	*D*—H⋯*A*
C17—H17*C*⋯O7^i^	0.96	2.42	3.346 (10)	162
C14—H14⋯O5^ii^	0.93	2.39	3.283 (11)	161
C17—H17*A*⋯O2^iii^	0.96	2.32	3.205 (11)	153
C16—H16⋯O2^iii^	0.93	2.39	3.159 (9)	140
C16—H16⋯O1^iii^	0.93	2.19	3.021 (2)	149
C13—H13*A*⋯O1^iii^	0.97	2.58	3.382 (3)	140
C17—H17*C*⋯O7^i^	0.96	2.42	3.346 (10)	162

Symmetry codes: (i) 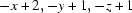; (ii) 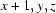; (iii) 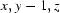.

**Fig. 1.**
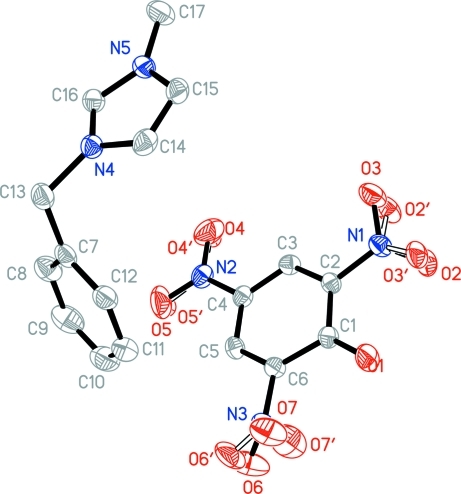
The structure of (I) showing the atom-numbering scheme. Displacement ellipsoids are drawn at the 30% probability level and H atoms have been omitted for clarity. Only the predominate nitro oxygen atoms are displayed for the disordered nitro groups [O2,O3,O4,O5 (0.54); O6,O7 (0.58)].

**Fig. 2.**
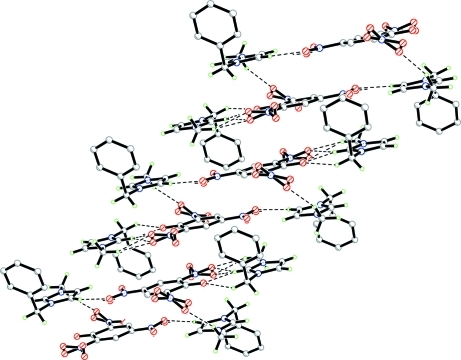
The molecular packing diagram of the title compound. Dashed lines indicate weak C—H···O hydrogen bonding interactions between the cation-anion pairs (Table 1). Both of the disordered oxygen atoms in the nitro groups of the picrate anions are displayed.

## supplementary crystallographic information

### Comment

Energetic materials are used extensively for both civilian and military applications (Sikder & Sikder, 2004). Heterocyclic organic salts with low melting points are a new class of energetic materials that has attracted considerable interest because of their "green chemistry" properties (Singh *et al.*, 2006). Picric acid is a polynitrogen compound with explosive character and imidazolium-based cation picrate salts are good candidates for energetic ionic salts (Jin *et al.*, 2005). Based on our continued interest in these compounds, the title organic salt (scheme 1) was prepared and its structure is reported.

The asymmetric unit of the title compound contains one independent cation (1-methyl-3-benzenylimidazolium)-anion (picrate) pair (Fig. 1). The dihedral angle between the benzene ring in the cation and the imidazolium ring and the benzene ring of the picrate anion is 113.7 (2)° and 116.3 (2) °, respectively. The imidazolium ring is nearly parallel to the benzene ring of the picrate anion with the dihedral angle of separation being 2.6 (1)°. The oxygen atoms in the nitro groups of the picrate anion are disorderd with the *o*-NO_2_ major components (O2, O3, O4, O5) being 0.54 occupied and the *p*-NO_2_major components (O5, O6) at 0.58 occupancy . Crystal packing is stabilized by the weak C—H···O interactions between the cation-anion pairs (Fig. 2, Table 1).

### Experimental

The title salt (C_11_H_13_N_2_)^+^.(C_6_H_2_N_3_O_7_)^-^ was synthesized using a slightly modified literature mothod (Jin *et al.*, 2005). It was crystallized by slow evaporation of an acetonitrile and methanol solution of the salt.

### Refinement

H atoms were positioned geometrically with C—H bond lengths fixed to 0.93 (aromatic CH),0.97 (methylene CH_2_) or 0.96Å (methyl CH_3_). A riding model was used during the refinement process. The *U*_iso_ parameters for H atoms were constrained to be 1.2*U*_eq_ of the carrier C atom for aromatic and methylene groups, and 1.5*U*_eq_ of the carrier C atom for methyl groups. Measured Friedel pairs were merged before refinement.

### Crystal data

**Table:**

C_11_H_13_N_2_^+^·C_6_H_2_N_3_O_7_^−^	*Z* = 2
*M**_r_* = 401.34	*F*(000) = 416
Triclinic, *P*1	*D*_x_ = 1.490 Mg m^−^^3^
Hall symbol: -P 1	Mo *K*α radiation, λ = 0.71073 Å
*a* = 9.1322 (6) Å	Cell parameters from 1924 reflections
*b* = 10.2060 (7) Å	θ = 2.2–26.5°
*c* = 10.8744 (7) Å	µ = 0.12 mm^−^^1^
α = 63.619 (1)°	*T* = 298 K
β = 80.166 (1)°	Block, yellow
γ = 86.482 (1)°	0.20 × 0.10 × 0.10 mm
*V* = 894.52 (10) Å^3^	

### Data collection

**Table:**

Bruker SMART APEX CCD area-detector diffractometer	3447 independent reflections
Radiation source: fine-focus sealed tube	2610 reflections with *I* > 2σ(*I*)
graphite	*R*_int_ = 0.046
φ and ω scans	θ_max_ = 26.0°, θ_min_ = 2.2°
Absorption correction: multi-scan (*SADABS*; Sheldrick, 1996)	*h* = −10→11
*T*_min_ = 0.986, *T*_max_ = 0.988	*k* = −12→12
5623 measured reflections	*l* = −11→13

### Refinement

**Table:**

Refinement on *F*^2^	Primary atom site location: structure-invariant direct methods
Least-squares matrix: full	Secondary atom site location: difference Fourier map
*R*[*F*^2^ > 2σ(*F*^2^)] = 0.056	Hydrogen site location: inferred from neighbouring sites
*wR*(*F*^2^) = 0.144	H-atom parameters constrained
*S* = 1.04	*w* = 1/[σ^2^(*F*_o_^2^) + (0.0707*P*)^2^] where *P* = (*F*_o_^2^ + 2*F*_c_^2^)/3
3447 reflections	(Δ/σ)_max_ < 0.001
320 parameters	Δρ_max_ = 0.35 e Å^−^^3^
15 restraints	Δρ_min_ = −0.24 e Å^−^^3^

### Special details

**Table:**

Geometry. All e.s.d.'s (except the e.s.d. in the dihedral angle between two l.s. planes) are estimated using the full covariance matrix. The cell e.s.d.'s are taken into account individually in the estimation of e.s.d.'s in distances, angles and torsion angles; correlations between e.s.d.'s in cell parameters are only used when they are defined by crystal symmetry. An approximate (isotropic) treatment of cell e.s.d.'s is used for estimating e.s.d.'s involving l.s. planes.
Refinement. Refinement of *F*^2^ against ALL reflections. The weighted *R*-factor *wR* and goodness of fit *S* are based on *F*^2^, conventional *R*-factors *R* are based on *F*, with *F* set to zero for negative *F*^2^. The threshold expression of *F*^2^ > σ(*F*^2^) is used only for calculating *R*-factors(gt) *etc*. and is not relevant to the choice of reflections for refinement. *R*-factors based on *F*^2^ are statistically about twice as large as those based on *F*, and *R*- factors based on ALL data will be even larger.

### Fractional atomic coordinates and isotropic or equivalent isotropic displacement parameters (Å^2^)

**Table:**

	*x*	*y*	*z*	*U*_iso_*/*U*_eq_	Occ. (<1)
C1	0.6669 (2)	0.9523 (2)	0.3757 (2)	0.0412 (5)	
C2	0.5960 (2)	0.8381 (2)	0.50638 (19)	0.0391 (5)	
C3	0.4985 (2)	0.7359 (2)	0.5166 (2)	0.0417 (5)	
H3	0.4582	0.6639	0.6035	0.050*	
C4	0.4602 (2)	0.7397 (2)	0.3977 (2)	0.0421 (5)	
C5	0.5191 (2)	0.8461 (2)	0.2678 (2)	0.0435 (5)	
H5	0.4912	0.8496	0.1881	0.052*	
C6	0.6182 (2)	0.9453 (2)	0.25820 (19)	0.0409 (5)	
C7	0.9029 (2)	0.4665 (2)	0.16740 (19)	0.0448 (5)	
C8	0.7605 (3)	0.4513 (3)	0.1476 (2)	0.0602 (6)	
H8	0.7217	0.3584	0.1754	0.072*	
C9	0.6758 (3)	0.5720 (4)	0.0873 (3)	0.0767 (8)	
H9	0.5803	0.5606	0.0741	0.092*	
C10	0.7316 (3)	0.7092 (3)	0.0466 (2)	0.0736 (8)	
H10	0.6740	0.7908	0.0058	0.088*	
C11	0.8721 (3)	0.7263 (3)	0.0659 (2)	0.0670 (7)	
H11	0.9097	0.8194	0.0390	0.080*	
C12	0.9572 (3)	0.6063 (2)	0.1247 (2)	0.0525 (6)	
H12	1.0532	0.6188	0.1362	0.063*	
C13	0.9942 (3)	0.3344 (2)	0.2325 (2)	0.0516 (6)	
H13A	0.9526	0.2525	0.2267	0.062*	
H13B	1.0944	0.3526	0.1807	0.062*	
C14	1.0841 (2)	0.3658 (2)	0.4261 (2)	0.0495 (5)	
H14	1.1477	0.4457	0.3713	0.059*	
C15	1.0570 (2)	0.2983 (2)	0.5651 (2)	0.0492 (5)	
H15	1.0988	0.3221	0.6249	0.059*	
C16	0.9239 (2)	0.1884 (2)	0.4886 (2)	0.0428 (5)	
H16	0.8581	0.1243	0.4855	0.051*	
C17	0.9006 (3)	0.0830 (3)	0.7460 (2)	0.0630 (7)	
H17A	0.8231	0.0235	0.7460	0.094*	
H17B	0.8617	0.1349	0.7993	0.094*	
H17C	0.9802	0.0218	0.7866	0.094*	
N1	0.6315 (2)	0.8244 (2)	0.63649 (18)	0.0492 (5)	
N2	0.3603 (2)	0.6289 (2)	0.4097 (2)	0.0525 (5)	
N3	0.6791 (2)	1.0516 (2)	0.11900 (19)	0.0537 (5)	
N4	1.00040 (18)	0.29513 (17)	0.37987 (16)	0.0416 (4)	
N5	0.95646 (18)	0.18786 (18)	0.60292 (16)	0.0436 (4)	
O1	0.75553 (19)	1.04635 (18)	0.36201 (16)	0.0646 (5)	
O2	0.6926 (10)	0.9248 (11)	0.6410 (15)	0.064 (2)	0.54
O3	0.6079 (6)	0.7030 (6)	0.7381 (6)	0.0693 (15)	0.54
O4	0.3127 (14)	0.5302 (14)	0.5267 (10)	0.091 (4)	0.54
O5	0.3362 (16)	0.6282 (15)	0.3029 (9)	0.093 (4)	0.54
O6	0.5945 (15)	1.1147 (16)	0.0366 (15)	0.104 (4)	0.58
O7	0.8115 (7)	1.0704 (12)	0.0890 (11)	0.075 (2)	0.58
O2'	0.5492 (7)	0.7504 (8)	0.7465 (7)	0.0700 (18)	0.46
O3'	0.7363 (11)	0.8962 (12)	0.6320 (17)	0.060 (2)	0.46
O5'	0.3250 (14)	0.6386 (14)	0.3006 (8)	0.061 (3)	0.46
O4'	0.3061 (11)	0.5414 (13)	0.5233 (7)	0.047 (2)	0.46
O6'	0.616 (2)	1.073 (2)	0.027 (2)	0.094 (5)	0.42
O7'	0.7953 (14)	1.1148 (18)	0.0970 (19)	0.114 (6)	0.42

### Atomic displacement parameters (Å^2^)

**Table:**

	*U*^11^	*U*^22^	*U*^33^	*U*^12^	*U*^13^	*U*^23^
C1	0.0384 (10)	0.0434 (11)	0.0450 (11)	−0.0048 (9)	−0.0101 (9)	−0.0205 (9)
C2	0.0363 (10)	0.0451 (11)	0.0391 (11)	0.0023 (9)	−0.0108 (8)	−0.0197 (9)
C3	0.0372 (11)	0.0410 (11)	0.0427 (11)	−0.0032 (9)	−0.0058 (9)	−0.0143 (9)
C4	0.0377 (10)	0.0424 (11)	0.0492 (12)	−0.0051 (9)	−0.0082 (9)	−0.0218 (10)
C5	0.0415 (11)	0.0514 (12)	0.0448 (11)	−0.0017 (9)	−0.0117 (9)	−0.0257 (10)
C6	0.0392 (11)	0.0431 (11)	0.0383 (10)	−0.0037 (9)	−0.0064 (8)	−0.0155 (9)
C7	0.0523 (12)	0.0520 (12)	0.0282 (10)	−0.0094 (10)	0.0025 (9)	−0.0177 (9)
C8	0.0590 (15)	0.0666 (16)	0.0465 (13)	−0.0155 (13)	−0.0010 (11)	−0.0180 (12)
C9	0.0572 (16)	0.106 (2)	0.0563 (16)	0.0023 (16)	−0.0112 (13)	−0.0260 (16)
C10	0.085 (2)	0.0757 (19)	0.0474 (14)	0.0179 (16)	−0.0074 (14)	−0.0192 (13)
C11	0.097 (2)	0.0537 (15)	0.0442 (13)	−0.0012 (14)	−0.0074 (13)	−0.0168 (11)
C12	0.0646 (14)	0.0521 (14)	0.0380 (11)	−0.0117 (11)	−0.0067 (10)	−0.0164 (10)
C13	0.0612 (14)	0.0543 (13)	0.0407 (11)	−0.0084 (11)	0.0008 (10)	−0.0242 (10)
C14	0.0472 (12)	0.0419 (12)	0.0629 (14)	−0.0066 (10)	−0.0119 (10)	−0.0242 (11)
C15	0.0530 (13)	0.0469 (12)	0.0599 (14)	0.0027 (10)	−0.0239 (11)	−0.0293 (11)
C16	0.0446 (11)	0.0424 (11)	0.0447 (11)	−0.0041 (9)	−0.0116 (9)	−0.0199 (9)
C17	0.0752 (16)	0.0644 (15)	0.0433 (13)	−0.0063 (13)	−0.0174 (11)	−0.0147 (11)
N1	0.0475 (11)	0.0565 (12)	0.0424 (10)	−0.0041 (9)	−0.0111 (8)	−0.0188 (9)
N2	0.0482 (11)	0.0498 (12)	0.0635 (13)	−0.0084 (9)	−0.0101 (11)	−0.0272 (11)
N3	0.0581 (13)	0.0584 (12)	0.0426 (11)	−0.0144 (10)	−0.0124 (10)	−0.0170 (9)
N4	0.0448 (9)	0.0405 (9)	0.0417 (9)	−0.0045 (8)	−0.0063 (7)	−0.0196 (8)
N5	0.0471 (10)	0.0443 (10)	0.0427 (10)	0.0013 (8)	−0.0156 (8)	−0.0191 (8)
O1	0.0735 (11)	0.0697 (11)	0.0521 (9)	−0.0361 (9)	−0.0076 (8)	−0.0242 (8)
O2	0.083 (5)	0.064 (4)	0.055 (3)	−0.012 (4)	−0.016 (4)	−0.032 (3)
O3	0.081 (4)	0.077 (4)	0.039 (2)	−0.019 (3)	−0.011 (3)	−0.013 (2)
O4	0.099 (7)	0.054 (5)	0.108 (7)	−0.019 (5)	−0.016 (5)	−0.023 (5)
O5	0.119 (8)	0.103 (7)	0.089 (8)	−0.022 (5)	−0.024 (5)	−0.066 (6)
O6	0.086 (4)	0.118 (8)	0.062 (5)	0.003 (5)	−0.028 (3)	0.005 (4)
O7	0.044 (2)	0.096 (5)	0.056 (3)	−0.021 (2)	0.0098 (19)	−0.011 (3)
O2'	0.068 (4)	0.094 (5)	0.040 (2)	−0.026 (3)	0.000 (3)	−0.022 (3)
O3'	0.064 (5)	0.067 (5)	0.058 (4)	−0.014 (4)	−0.021 (4)	−0.031 (3)
O5'	0.063 (5)	0.059 (5)	0.058 (6)	−0.037 (4)	−0.017 (4)	−0.014 (4)
O4'	0.048 (4)	0.053 (5)	0.039 (4)	−0.031 (4)	0.005 (3)	−0.018 (4)
O6'	0.127 (11)	0.104 (9)	0.047 (4)	−0.047 (7)	−0.034 (6)	−0.017 (5)
O7'	0.126 (9)	0.122 (11)	0.080 (5)	−0.060 (8)	−0.017 (6)	−0.026 (6)

### Geometric parameters (Å, °)

**Table:**

C1—O1	1.235 (2)	C13—H13B	0.9700
C1—C2	1.451 (3)	C14—C15	1.336 (3)
C1—C6	1.454 (3)	C14—N4	1.371 (3)
C2—C3	1.368 (3)	C14—H14	0.9300
C2—N1	1.450 (2)	C15—N5	1.367 (3)
C3—C4	1.379 (3)	C15—H15	0.9300
C3—H3	0.9300	C16—N4	1.319 (2)
C4—C5	1.383 (3)	C16—N5	1.325 (2)
C4—N2	1.443 (3)	C16—H16	0.9300
C5—C6	1.357 (3)	C17—N5	1.464 (3)
C5—H5	0.9300	C17—H17A	0.9600
C6—N3	1.451 (3)	C17—H17B	0.9600
C7—C8	1.383 (3)	C17—H17C	0.9600
C7—C12	1.385 (3)	N1—O2	1.220 (9)
C7—C13	1.493 (3)	N1—O3'	1.222 (10)
C8—C9	1.373 (4)	N1—O2'	1.235 (7)
C8—H8	0.9300	N1—O3	1.240 (6)
C9—C10	1.369 (4)	N2—O4'	1.197 (7)
C9—H9	0.9300	N2—O5	1.222 (9)
C10—C11	1.368 (4)	N2—O5'	1.243 (9)
C10—H10	0.9300	N2—O4	1.245 (9)
C11—C12	1.367 (3)	N3—O6'	1.168 (11)
C11—H11	0.9300	N3—O7	1.201 (7)
C12—H12	0.9300	N3—O7'	1.208 (11)
C13—N4	1.480 (3)	N3—O6	1.215 (10)
C13—H13A	0.9700		
			
O1—C1—C2	126.06 (18)	N4—C14—H14	126.5
O1—C1—C6	122.83 (18)	C14—C15—N5	107.38 (18)
C2—C1—C6	111.10 (16)	C14—C15—H15	126.3
C3—C2—N1	116.05 (17)	N5—C15—H15	126.3
C3—C2—C1	124.07 (18)	N4—C16—N5	108.52 (17)
N1—C2—C1	119.85 (17)	N4—C16—H16	125.7
C2—C3—C4	119.81 (18)	N5—C16—H16	125.7
C2—C3—H3	120.1	N5—C17—H17A	109.5
C4—C3—H3	120.1	N5—C17—H17B	109.5
C3—C4—C5	120.79 (18)	H17A—C17—H17B	109.5
C3—C4—N2	119.28 (18)	N5—C17—H17C	109.5
C5—C4—N2	119.92 (19)	H17A—C17—H17C	109.5
C6—C5—C4	119.12 (19)	H17B—C17—H17C	109.5
C6—C5—H5	120.4	O2—N1—O2'	112.3 (8)
C4—C5—H5	120.4	O3'—N1—O2'	122.1 (8)
C5—C6—N3	116.53 (18)	O2—N1—O3	122.5 (7)
C5—C6—C1	125.09 (18)	O3'—N1—O3	116.7 (8)
N3—C6—C1	118.38 (17)	O2—N1—C2	120.6 (7)
C8—C7—C12	118.2 (2)	O3'—N1—C2	118.2 (8)
C8—C7—C13	120.1 (2)	O2'—N1—C2	119.5 (4)
C12—C7—C13	121.7 (2)	O3—N1—C2	116.6 (3)
C9—C8—C7	120.6 (2)	O4'—N2—O5	123.1 (7)
C9—C8—H8	119.7	O4'—N2—O5'	123.6 (5)
C7—C8—H8	119.7	O5—N2—O4	122.0 (7)
C10—C9—C8	120.2 (3)	O5'—N2—O4	123.2 (7)
C10—C9—H9	119.9	O4'—N2—C4	118.7 (4)
C8—C9—H9	119.9	O5—N2—C4	118.2 (5)
C11—C10—C9	120.1 (3)	O5'—N2—C4	117.4 (4)
C11—C10—H10	120.0	O4—N2—C4	119.5 (6)
C9—C10—H10	120.0	O6'—N3—O7	115.7 (13)
C12—C11—C10	119.9 (2)	O6'—N3—O7'	120.1 (12)
C12—C11—H11	120.0	O7—N3—O6	122.8 (9)
C10—C11—H11	120.0	O7'—N3—O6	115.6 (13)
C11—C12—C7	121.1 (2)	O6'—N3—C6	119.1 (10)
C11—C12—H12	119.5	O7—N3—C6	118.5 (5)
C7—C12—H12	119.5	O7'—N3—C6	120.8 (9)
N4—C13—C7	112.48 (16)	O6—N3—C6	118.6 (8)
N4—C13—H13A	109.1	C16—N4—C14	108.63 (17)
C7—C13—H13A	109.1	C16—N4—C13	125.71 (16)
N4—C13—H13B	109.1	C14—N4—C13	125.65 (17)
C7—C13—H13B	109.1	C16—N5—C15	108.46 (17)
H13A—C13—H13B	107.8	C16—N5—C17	126.17 (18)
C15—C14—N4	107.00 (18)	C15—N5—C17	125.31 (18)
C15—C14—H14	126.5		
			
O1—C1—C2—C3	−179.9 (2)	C3—C2—N1—O2'	−18.1 (4)
C6—C1—C2—C3	1.3 (3)	C1—C2—N1—O2'	164.1 (4)
O1—C1—C2—N1	−2.2 (3)	C3—C2—N1—O3	20.3 (4)
C6—C1—C2—N1	178.93 (17)	C1—C2—N1—O3	−157.5 (3)
N1—C2—C3—C4	−179.13 (17)	C3—C4—N2—O4'	4.4 (8)
C1—C2—C3—C4	−1.4 (3)	C5—C4—N2—O4'	−177.1 (7)
C2—C3—C4—C5	0.0 (3)	C3—C4—N2—O5	−174.9 (10)
C2—C3—C4—N2	178.38 (18)	C5—C4—N2—O5	3.6 (10)
C3—C4—C5—C6	1.5 (3)	C3—C4—N2—O5'	177.9 (9)
N2—C4—C5—C6	−176.96 (18)	C5—C4—N2—O5'	−3.6 (9)
C4—C5—C6—N3	178.34 (19)	C3—C4—N2—O4	−1.4 (8)
C4—C5—C6—C1	−1.5 (3)	C5—C4—N2—O4	177.0 (8)
O1—C1—C6—C5	−178.7 (2)	C5—C6—N3—O6'	19.6 (10)
C2—C1—C6—C5	0.2 (3)	C1—C6—N3—O6'	−160.5 (10)
O1—C1—C6—N3	1.4 (3)	C5—C6—N3—O7	−130.6 (6)
C2—C1—C6—N3	−179.67 (17)	C1—C6—N3—O7	49.3 (7)
C12—C7—C8—C9	−0.1 (3)	C5—C6—N3—O7'	−158.5 (9)
C13—C7—C8—C9	−179.7 (2)	C1—C6—N3—O7'	21.4 (9)
C7—C8—C9—C10	−0.3 (4)	C5—C6—N3—O6	47.4 (7)
C8—C9—C10—C11	0.0 (4)	C1—C6—N3—O6	−132.7 (7)
C9—C10—C11—C12	0.6 (4)	N5—C16—N4—C14	−0.4 (2)
C10—C11—C12—C7	−1.0 (3)	N5—C16—N4—C13	−178.99 (17)
C8—C7—C12—C11	0.7 (3)	C15—C14—N4—C16	0.5 (2)
C13—C7—C12—C11	−179.68 (19)	C15—C14—N4—C13	179.14 (19)
C8—C7—C13—N4	−102.8 (2)	C7—C13—N4—C16	102.4 (2)
C12—C7—C13—N4	77.6 (2)	C7—C13—N4—C14	−76.0 (2)
N4—C14—C15—N5	−0.4 (2)	N4—C16—N5—C15	0.1 (2)
C3—C2—N1—O2	−165.4 (5)	N4—C16—N5—C17	−177.20 (19)
C1—C2—N1—O2	16.8 (5)	C14—C15—N5—C16	0.2 (2)
C3—C2—N1—O3'	167.5 (6)	C14—C15—N5—C17	177.5 (2)
C1—C2—N1—O3'	−10.3 (6)		

### Hydrogen-bond geometry (Å, °)

**Table:**

*D*—H···*A*	*D*—H	H···*A*	*D*···*A*	*D*—H···*A*
C17—H17C···O7^i^	0.96	2.42	3.346 (10)	162
C14—H14···O5^ii^	0.93	2.39	3.283 (11)	161
C17—H17A···O2^iii^	0.96	2.32	3.205 (11)	153
C16—H16···O2^iii^	0.93	2.39	3.159 (9)	140
C16—H16···O1^iii^	0.93	2.19	3.021 (2)	149
C13—H13A···O1^iii^	0.97	2.58	3.382 (3)	140
C17—H17C···O7^i^	0.96	2.42	3.346 (10)	162

Symmetry codes: (i) −*x*+2, −*y*+1, −*z*+1; (ii) *x*+1, *y*, *z*; (iii) *x*, *y*−1, *z*.
